# Spatio-temporal evolution and trend prediction of the incidence of Class B notifiable infectious diseases in China: a sample of statistical data from 2007 to 2020

**DOI:** 10.1186/s12889-022-13566-2

**Published:** 2022-06-17

**Authors:** Ruo-Nan Wang, Yue-Chi Zhang, Bo-Tao Yu, Yan-Ting He, Bei Li, Yi-Li Zhang

**Affiliations:** 1 School of Health Management, Southern Medical University, Guangzhou, 510515 China; 2 Bussiness School, University of Aberdeen, Aberdeen, UK

**Keywords:** Class B notifiable infectious diseases, Incidence, Spatial and temporal evolution, Trend prediction

## Abstract

**Background:**

With the accelerated global integration and the impact of climatic, ecological and social environmental changes, China will continue to face the challenge of the outbreak and spread of emerging infectious diseases and traditional ones. This study aims to explore the spatial and temporal evolutionary characteristics of the incidence of Class B notifiable infectious diseases in China from 2007 to 2020, and to forecast the trend of it as well. Hopefully, it will provide a reference for the formulation of infectious disease prevention and control strategies.

**Methods:**

Data on the incidence rates of Class B notifiable infectious diseases in 31 provinces, municipalities and autonomous regions of China from 2007 to 2020 were collected for the prediction of the spatio-temporal evolution and spatial correlation as well as the incidence of Class B notifiable infectious diseases in China based on global spatial autocorrelation and Autoregressive Integrated Moving Average (ARIMA).

**Results:**

From 2007 to 2020, the national incidence rate of Class B notifiable infectious diseases (from 272.37 per 100,000 in 2007 to 190.35 per 100,000 in 2020) decreases year by year, and the spatial distribution shows an “east-central-west” stepwise increase. From 2007 to 2020, the spatial clustering of the incidence of Class B notifiable infectious diseases is significant and increasing year by year (Moran’s *I* index values range from 0.189 to 0.332, *p <* 0.05). The forecasted incidence rates of Class B notifiable infectious diseases nationwide from 2021 to 2024 (205.26/100,000, 199.95/100,000, 194.74/100,000 and 189.62/100,000) as well as the forecasted values for most regions show a downward trend, with only some regions (Guangdong, Hunan, Hainan, Tibet, Guangxi and Guizhou) showing an increasing trend year by year.

**Conclusions:**

The current study found that since there were significant regional disparities in the prevention and control of infectious diseases in China between 2007 and 2020, the reduction of the incidence of Class B notifiable infectious diseases requires the joint efforts of the surrounding provinces. Besides, special attention should be paid to provinces with an increasing trend in the incidence of Class B notifiable infectious diseases to prevent the re-emergence of certain traditional infectious diseases in a particular province or even the whole country, as well as the outbreak and spread of emerging infectious diseases.

**Supplementary Information:**

The online version contains supplementary material available at 10.1186/s12889-022-13566-2.

## Background

The occurrence and transmission patterns of infectious diseases are changing with the accelerated global integration and the impact of climatic, ecological and social environmental changes [1, 2]. Recent outbreaks of infectious diseases such as Ebola, Zika and Corona Virus Disease 2019 (COVID-19) demonstrate that emerging and traditional infectious diseases are posing and will continue to pose a threat to human life and health, which brings new challenges for the emergency response capacity of the whole world, especially developing countries with limited available resources [3–5]. Since the outbreak of the Severe acute respiratory syndrome (SARS) in 2003, China, as the world’s largest developing country, has made many efforts to build a response system for public health emergencies, promulgating a number of emergency plans, overhauling the national information system for disease prevention and control, and launching National Major Scientific and Technological Special Project for Prevention and Control. Yet the latest epidemic profile of notifiable infectious diseases released by the Chinese Centre for Disease Control and Prevention show that the number of reported cases of notifiable infectious diseases in mainland China remained as many as 699,466, and that 1531 people died between 00:00 on 1 May 2021 and 24:00 on 31 May 2021 [6]. Thus China still remains under serious threat from infectious diseases.

The primary task for controlling infectious diseases is to understand the distribution characteristics of epidemiology. According to the existing research, about 80% of epidemiological data are spatial in nature [7]. Therefore, the exact analysis of the spatial distribution characteristics of infectious diseases is a must for the effective study of the causes and other influencing factors of diseases and also for the formulation of effective prevention and control strategies. In recent years, with a mature statistical method, spatio-temporal statistics, researchers can not only conduct a dynamic analysis of the temporal and spatial distribution characteristics of infectious diseases, but also summarize the spatio-temporal transmission patterns by considering the three-dimensional environment in which they occur and are prevalent [8, 9]. So far, extensive studies of the epidemiological characteristics of infectious diseases have been conducted by many scholars employing spatial statistical methods based on geographic information system (GIS) [10–14].

Modelling spatio-temporal trends in infectious diseases is of great importance, because real-time epidemiological forecasting can help to predict the geographic expansion of diseases as well as the number of cases. What’s more, the exact prediction of the epidemic outbreak helps policy makers to prepare early so that they can better implement public health interventions. As the infectivity of pathogens and the availability of drugs and vaccines change over time, the application of updated shared data is much necessary for the evaluation and prediction of disease hazard. In China, the National Health Commission of the People’s Republic of China regularly releases on its official website the annual incidence of Class A, Class B and Class C notifiable infectious diseases, providing a platform for further study of the epidemiological characteristics of notifiable infectious diseases in China. In the Law of the PRC on the Prevention and Treatment of Infectious Diseases enacted in 1989, infectious diseases are classified into three categories, Class A, Class B and Class C, among which the former two can both cause large-scale severe epidemics within a short period of time, while the third is less infectious and causes only minor outbreaks. The number of notifiable infectious diseases in the three categories is ever-changing with the outbreak of emerging ones. For example, on October 2, 2020, the National Health Commission issued a draft of the revised Law on Prevention and Control of Infectious Diseases for consultation, which clearly states that two new types of infectious diseases, namely human H7N9 avian influenza and novel coronavirus, have been added to Class B. Currently, the three categories contain altogether 41 notifiable infectious diseases, as shown in Additional file 1: Annex 1. Epidemic incidence data have been shown to be valuable epidemiological tools for real-time assessment and prediction of trends and transmission potential [15–18]. By means of the data, predictive models can be used to help provide timely forecasts of disease incidence and geographic spread of emerging epidemics. Auto Regressive Integrated Moving Average (ARIMA) is a time-domain tool for time series analysis that has been widely used for infectious disease prediction [19, 20]. For example, many scholars have recently used ARIMA models to predict the incidence of COVID-19 [21–23], as it was used to predict the incidence of other infectious diseases such as viral hepatitis [24], malaria [25, 26] and measles [27].

Despite the accumulated findings, there are still limitations in the existing research. In general, most of the current researches on infectious diseases are the study of one specific disease, and the hotspots of research have been focused on a single study of the current epidemiological status, prediction of incidence trends and spatial attributes of the disease, lacking systematic research on the epidemiological characteristics and trend prediction of multi-species combinations. Since there is growing evidence of the complexity of disease interactions and disease etiology, a multi-species analysis to capture the overall trends of infectious diseases may be a worthwhile approach. Therefore, by using the method of spatial econometric analysis and time series forecasting combined and taking 31 Chinese provinces (Hong Kong, Macao and Taiwan are excluded) as the study unit, this study aims to focus on the current spatial and temporal distribution of the incidence of highly infectious Class B notifiable infectious diseases, and to predict the future development trend of the incidence of Class B notifiable infectious diseases. Hopefully, it will help Chinese public policy makers to formulate better health policy intervention measures, build better response of China’s public health epidemic prevention system to disease outbreaks, and provide for other countries valuable information to develop better intervention strategies to prevent and control the spread of emerging and re-emerging infectious diseases.

## Data and methods

### Data sources

Firstly, with Class B notifiable infectious diseases as the research object, only data on the incidence of Class B were collected, because Class C require only appropriate surveillance since they do not cause any serious consequences, and Class A, which include only two types of infectious diseases, i.e. plague and cholera, are relatively few in number and have been almost eradicated in China. Secondly, given the availability of data, the data on the incidence of Class B notifiable infectious diseases used in this study were that of 31 provinces in mainland China (except Hong Kong, Macao and Taiwan) and that of the whole country for each year during the period 2007–2020. The data used in this study were obtained from public sources: (1) Data on the incidence of Class B notifiable infectious diseases were obtained from the China Health Statistical Yearbook 2008–2012 and the China Health and Family Planning Statistical Yearbook 2013–2021 published by the National Health Commission of the People’s Republic of China (http://www.nhc.gov.cn). (2) Geographic information based data were obtained from the standard map service website of the National Bureau of Surveying, Mapping and Geographic Information (http://bzdt.ch.mnr.gov.cn).

### Research methodology

#### Global spatial autocorrelation [28]

Global spatial autocorrelation analysis is performed at the national macro level by comparing the mean values of attributes aggregated over the overall region with the values of attributes on each spatial unit to derive the average degree of association between the incidence of Class B notifiable infectious diseases in each province and region at the national level, i.e. to determine whether there is any clustering of the incidence of Class B notifiable infectious diseases at the national level. Global spatial autocorrelation analysis usually uses the Moran’s *I* index to determine whether the spatial distribution of regional variables is statistically clustered or dispersed, and is calculated using the formula:

$$\mathrm{I}=\frac{\mathrm{n}\sum \limits_{\mathrm{i}=1}^\mathrm{n}\sum \limits_{\mathrm{j}=1}^\mathrm{n}{\mathrm{W}}_{\mathrm{ij}}\left({\mathrm{x}}_\mathrm{i}-\overline{\mathrm{x}}\right)\kern0.24em \left({\mathrm{x}}_\mathrm{j}-\overline{\mathrm{x}}\right)}{\sum \limits_{\mathrm{i}=1}^\mathrm{n}\sum \limits_{\mathrm{j}=1}^\mathrm{n}{\mathrm{W}}_{\mathrm{ij}}\sum \limits_{\mathrm{i}=1}^\mathrm{n}{\left({\mathrm{x}}_\mathrm{i}-\overline{\mathrm{x}}\right)}^2}$$

In the formula: *n* is the number of provincial administrative districts; *x*_*i*_ is the incidence of Class B notifiable infectious diseases in the *i*th provincial administrative district; *W*_*ij*_ is the spatial weight matrix, which is determined here by means of spatial geographical adjacency, i.e. if two places are adjacent, the corresponding element in the matrix takes 1, otherwise it takes 0; $\overline{x}$ is the mean incidence rate of Class B notifiable infectious diseases aggregated across all *n* provincial administrative districts.

Moran’s *I* index values generally range from [− 1,1]. At a given level of significance, *I >* 0 indicates a positive spatial correlation, with larger values indicating more pronounced spatial aggregation; *I <* 0 indicates a negative spatial correlation, with smaller values indicating more pronounced spatial dispersion; and *I =* 0 indicates that the observations are randomly distributed in space.

#### ARIMA model [29]

The ARIMA model is one of the most commonly used methods for infectious disease time series forecasting and has been shown to be highly accurate [30]. In ARIMA (p, d, q), AR(p) is an autoregressive model and MA(q) is a moving average model, with p, d and q being the number of autoregressive terms, the number of differences and the number of moving average terms respectively. The ARIMA (p, d, q) model is an autoregressive moving average process with smoothness obtained after *d* differences of the AR(p) process and the MA(q) process.

ARIMA modelling includes smooth constructive simulation fitting, parameter estimation and model diagnosis. The modelling process in this study consists of three main steps. Firstly, as the annual data used in this study did not show seasonal variation and were non-stationary, the series were differenced at the non-seasonal level, and the autocorrelation and partial autocorrelation plots were plotted to determine whether the differenced time series were stationary. Secondly, the best model was screened using the Bayesian Information Criterion Error (BIC) (the smaller the value, the better the model) based on the Box-Ljung test that the residual series were white noise. Finally, the accuracy of the model was evaluated in two ways. On the one hand, the fit of the ARIMA model between the actual and predicted values was determined by observing whether the actual values were within the 95% confidence interval of the predicted values. On the other hand, the accuracy of the ARIMA model was evaluated by calculating the relative error (RE) and the mean absolute percentage error (MAPE). The prediction accuracy of the model is generally considered to be high when the MAPE is < 10%. RE and MAPE are calculated as $\mathrm{RE}=\left|\frac{{\hat{y}}_t}{y_t}-1\right|\times 100\%$ and $\mathrm{MAPE}=\frac{100\%}{n}\sum \limits_{t=1}^n\left|\frac{{\hat{y}}_t}{y_t}-1\right|$, where the equations *y*_*t*_ and ${\hat{y}}_t$ denote the actual and predicted values at time point respectively.

As COVID-19 epidemic broke out in China in 2020, the 2020 data for Class B notifiable infectious diseases may affect the selection of the best model and in turn, the accuracy of the prediction results. Therefore, we first used 2007–2019 data for the above model construction for accurate forecast, followed by verifying the reliability of the best model selected in the above modelling process with 2020 data. If the relative error between the forecasted and actual incidence of Class B notifiable infectious diseases in 2020 is still less than 10%, it proves the reliability of the best model selected, which will continue to be used for short-term forecasting of the incidence of Class B notifiable infectious diseases.

Spatial correlation analysis in this study was performed using Stata 14.0 software; ARIMA model prediction using SPSS 26.0 software and mapping using ArcGIS 10.2 software. α = 0.05 was used as the significance criterion.

## Results

### Analysis of the spatial and temporal evolution of the incidence of Class B notifiable infectious diseases in China

#### Temporal evolutionary characteristics

**Fig. 1:**
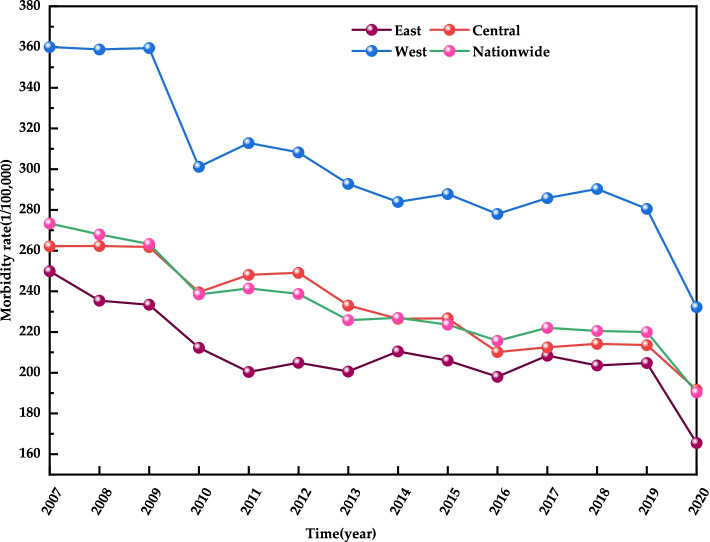
Regional incidence rates of Class B notifiable infectious diseases, China, 2007–2020

In order to study the trends in the incidence rates of Class B notifiable infectious diseases in different regions of China, this study divided the 31 provinces of China into three regions (east, central and west) in the light of economic development level and geographical location, with the east the highest in the economic development level while the west the lowest [31]. Figure 1 shows the trend of the incidence rate of Class B notifiable infectious diseases in this study in order to analyse regional differences in the incidence rate of infectious diseases in China. From 2007 to 2020, China’s national incidence of Class B notifiable infectious diseases shows a decreasing trend year by year (from 272.37/100,000 in 2007 to 190.35/100,000 in 2020), with the east dropping from 249.93/100,000 in 2007 to 165.45/100,000 in 2020, the central from 262.18/100,000 in 2007 to 191.63/100,000 in 2020 and the west from 360.07/100,000 in 2007 to 232.18/100,000 in 2020. The national incidence of Class B notifiable infectious diseases is on a gradual increase from the east to the central and then to the west, with the east the lowest incidence of Class B notifiable infectious diseases, while the west the highest. As for the decreasing range, the biggest is in the west (35.52%), followed by the east (33.80%) and the central (26.91%).

#### Spatial distribution characteristics

**Fig. 2:**
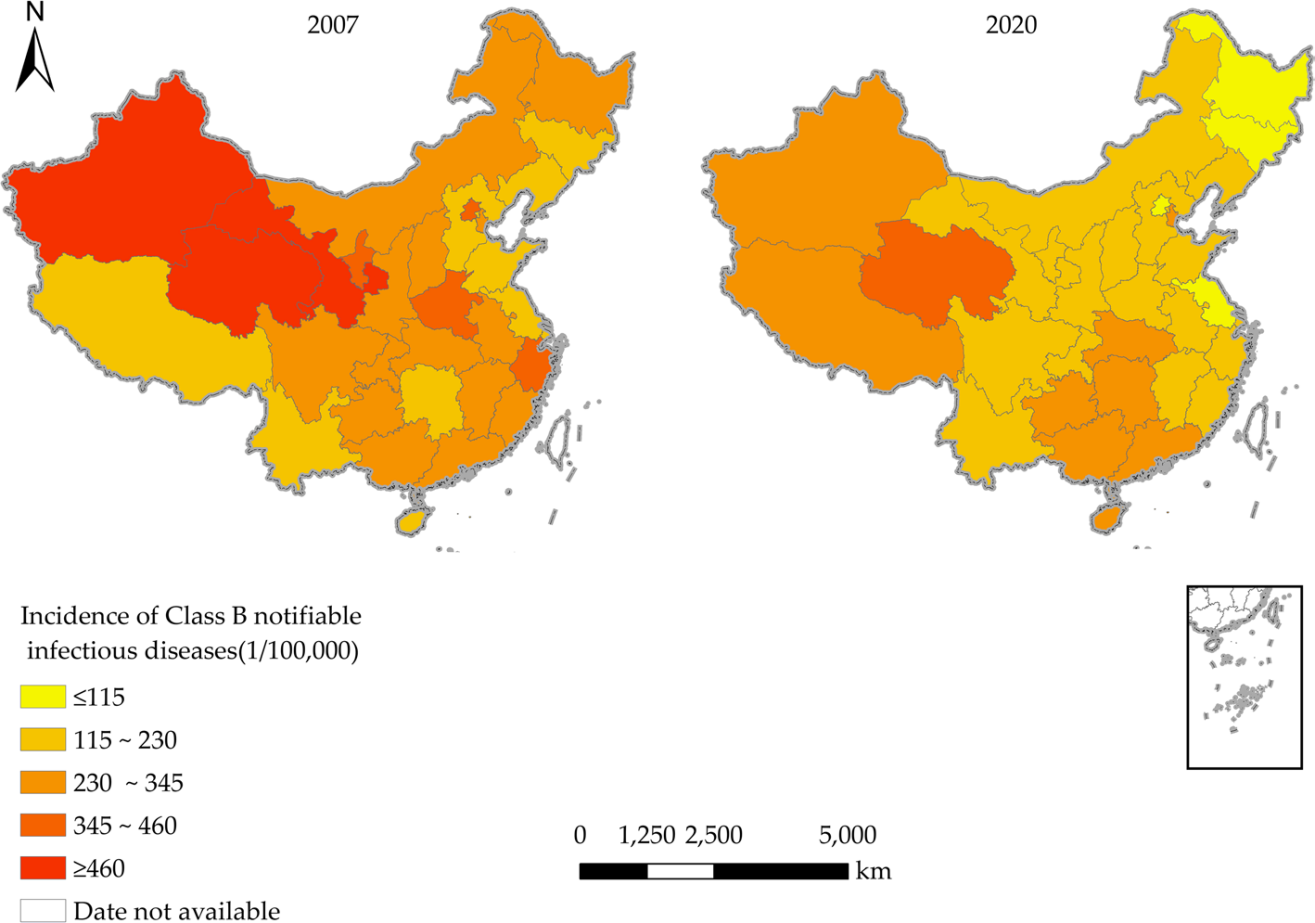
Provincial distribution of the incidence of Class B notifiable infectious diseases, 2007 and 2020

The incidence data of Class B notifiable infectious diseases in 2007 and 2020 in China were selected and visualized. Besides, in order to show the differences between regions more clearly, the same interval method in ArcGIS 10.2 software was used to classify the incidence into five classes: low (0/100,000~), slightly low (115/100,000~), medium (230/100,000~), slightly high (345/100,000~) and high (460/100,000~), see Fig. 2. In terms of spatial distribution, the incidence rate of Class B notifiable infectious diseases in each province has obvious spatial variation, with an overall increasing distribution of “east-central-west”, showing a clear correlation with the level of economic development. In the economically developed eastern region, with the exception of a few provinces, the incidence rates of Class B notifiable infectious diseases are all at or below the lower end of the provincial incidence rankings. For example, the incidence rates of Class B notifiable infectious diseases in Jiangsu Province in 2007 and 2020 were 172.3 per 100,000 and 98.23 per 100,000 respectively, ranking 30th and 29th among all the provinces. Compared with the east and central regions, the incidence rates of Class B notifiable infectious diseases in the less developed western regions are relatively high. For example, the levels of Class B notifiable infectious diseases in Xinjiang (657.52/100,000) and Qinghai (463.63/100,000) in 2007 and Qinghai (376.80/100,000) and Xinjiang (324.99/100,000) in 2020 are all at the higher and above, ranking in the top four among provinces. Compared with 2007, the incidence rates of Class B notifiable infectious diseases decreased in all provinces in 2020, except Guangdong, Hainan, Hunan and Tibet, where the incidence rates of Class B notifiable infectious diseases increased.

### Spatial autocorrelation analysis of the incidence of Class B notifiable infectious diseases in China

**Fig. 3:**
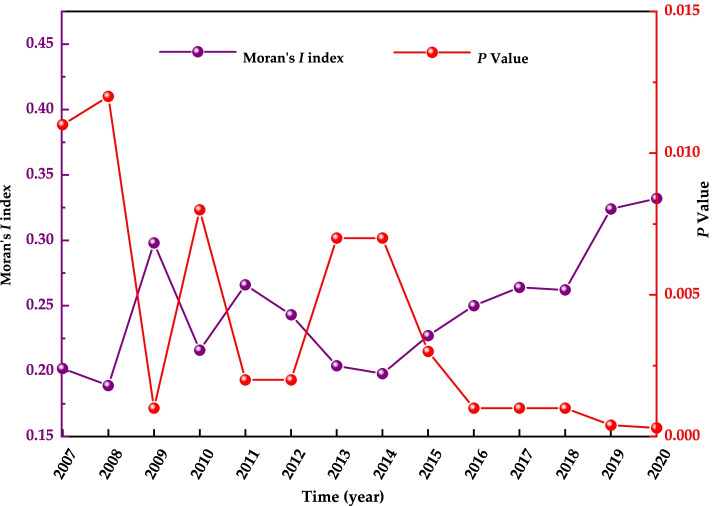
Moran’s *I* index chart of the incidence of Class B notifiable infectious diseases, 2007–2020

Figure 3 shows the trend of Moran’s *I* index for the incidence of Class B notifiable infectious diseases in China during the period 2007–2020, derived from the global spatial autocorrelation analysis of this study. The Moran’s *I* index values for the incidence of Class B notifiable infectious diseases in China during the period 2007–2020 ranged from 0.189 to 0.332, with an overall increasing trend and reaching significance levels in all years (*P <* 0.05) (see Fig. 3).

### Predicting the incidence of Class B notifiable infectious diseases in China

**Fig. 4:**
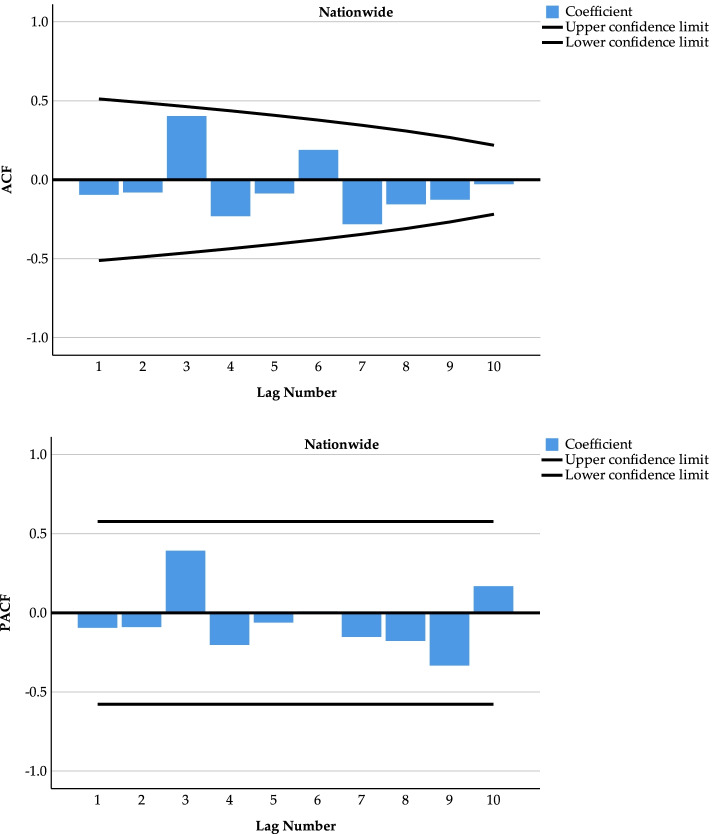
ACF and PACF plots of the national incidence of Class B notifiable infectious diseases after first-order differencing

The national incidence rate of Class B notifiable infectious diseases was differenced 1 time (*d*=1) to obtain an autocorrelation function (ACF) plot and a partial autocorrelation function (PACF) plot (see Fig. 4). The results show that the confidence intervals are within the range of [− 0.5,0.5], so the series after the first-order difference tends to be smooth.

The ARIMA model was fitted with the national incidence rate of Class B notifiable infectious diseases as the dependent variable, and the model was screened by Box-Ljung test and BIC index. Considering that p and q values generally did not exceed 2, a trial subscription from 0 to 2 was executed. Only five models passed the Box-Ljung test (*P >* 0.05): ARIMA (0, 1, 1), ARIMA (0, 1, 2), ARIMA (1, 1, 1), ARIMA (1, 1, 0), and ARIMA (2, 1, 1). Table 1 shows the statistics, BIC and parameter estimation results for the five models. The ARIMA (1, 1, 1) model was finally chosen as the optimal model with BIC = 4.631, standard R^2^ = 0.543 and MAPE = 2.169%.

**Table 1:** Parameter estimation and model validation of the ARIMA model

Models	Fitted Model Statistics			Ljung-Box Q(18)	
	Stationary R^**2**^	MAPE	BIC	Statistics	Sig.
ARIMA(0,1,1)	0.47	2.358	4.831	14.469	0.481
ARIMA(0,1,2)	0.219	3.427	5.983	21.53	0.198
ARIMA(1,1,1)	0.543	2.169	4.631	15.472	0.378
ARIMA(1,1,0)	0.453	2.523	4.934	17.571	0.329
ARIMA(2,1,1)	0.273	2.743	5.778	20.678	0.217

**Table 2:** National ARIMA model fitting results for the incidence of Class B notifiable infectious diseases

Year	Actual value	Forecast	Relative error (%)
2007	272.37	272.47	0.04
2008	267.93	269.68	0.65
2009	263.29	264.69	0.53
2010	238.47	251.91	5.64
2011	241.44	237.75	1.53
2012	238.75	237.78	0.41
2013	225.8	232.02	2.75
2014	226.97	221.99	2.19
2015	223.6	223.24	0.16
2016	215.68	219.68	1.85
2017	222.06	208.09	6.29
2018	220.51	214.99	2.50
2019	219.98	211.92	3.66

Table 2 shows the results of the fit using the ARIMA (1,1,1) model, which shows that the RE values of the data for each period is less than 10%, indicating that the model fits well.

**Fig. 5:**
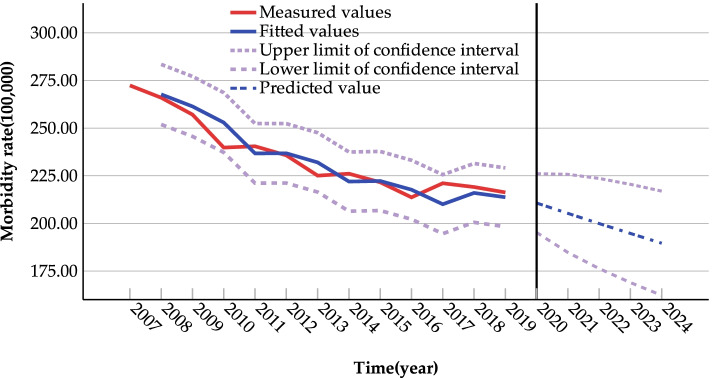
ARIMA model’s backgeneration and prediction of national incidence of Class B notifiable infectious diseases

Figure 5 shows the backgeneration and prediction of the national incidence of Class B notifiable infectious diseases using the ARIMA (1,1,1) model. According to Fig. 5, the actual values are within the 95% confidence interval of the predicted values, a further indication that the model fits well. The reliability of the ARIMA (1,1,1) model was verified using the data for 2020, and the RE value between the predicted value (207.67/100,000) and the actual value (190.35/100,000) for 2020 was 8.34% (RE < 10%), which indicates that the model has a good prediction effect. By comparing the predicted value with the actual value in 2020, we can find that the predicted value is larger than the actual value. The predicted incidence rates of Class B notifiable infectious diseases for 2021–2024 are 205.26/100,000, 199.95/100,000, 194.74/100,000 and 189.62/100,000 respectively, showing a decreasing trend year by year, as shown in Fig. 5.

**Fig. 6:**
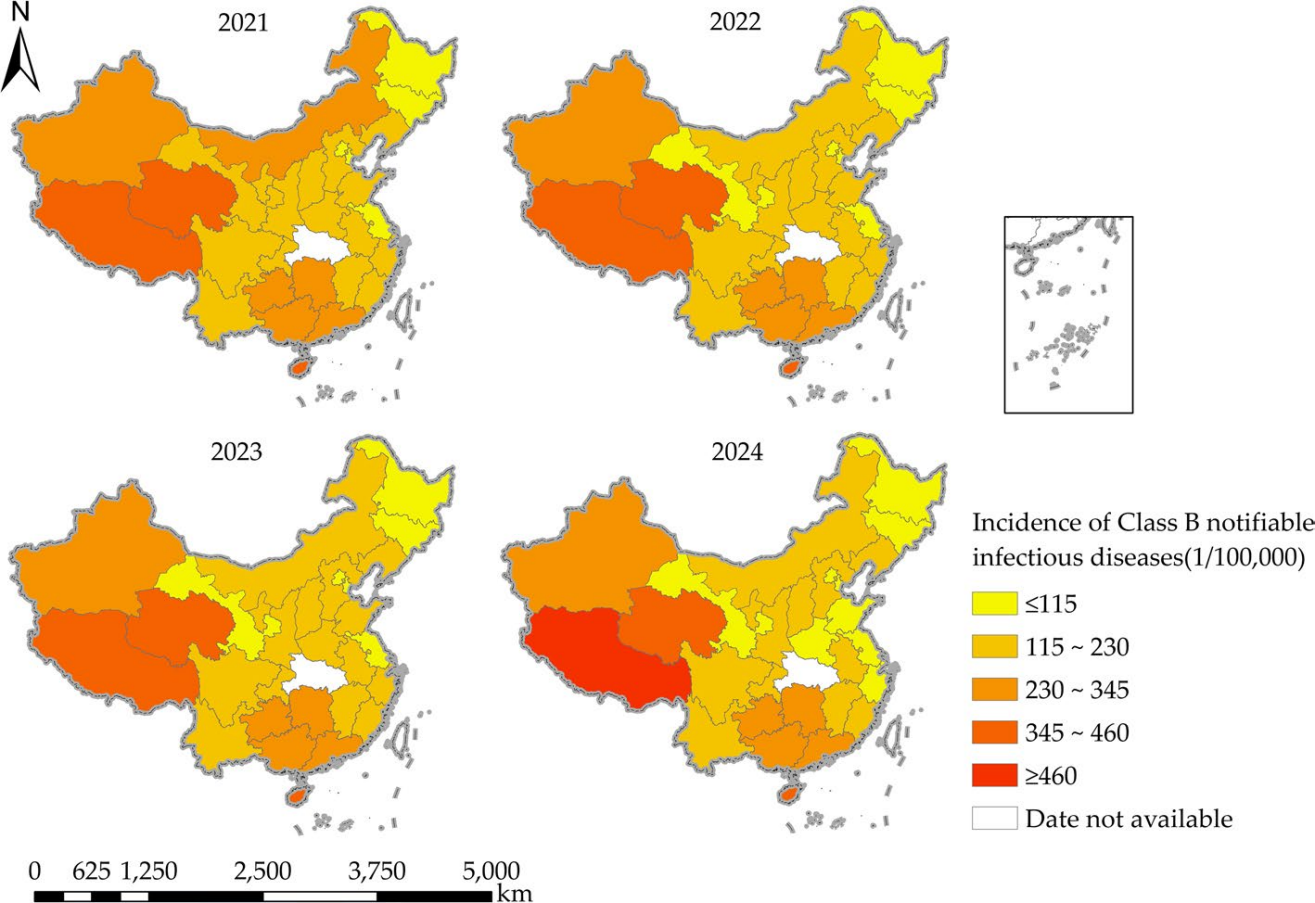
Spatial distribution of the predicted incidence of Class B notifiable infectious diseases, 2021–2024

The ARIMA model was used to fit the incidence rates of Class B notifiable infectious diseases in 31 provinces, municipalities and autonomous regions of China from 2007 to 2019, and the MAPE values for all provinces were less than 10%. Meanwhile, the results of the backgeneration validation using 2020 data showed that the RE values between the predicted and actual values in 2020 were also less than 10% for all the provinces except Hubei, indicating good fitting results for each province. See Table 3. It is worth mentioning that Hubei Province, as the initial outbreak place of the epidemic in China, may have been hit so hard by the epidemic that the best model selected did not pass the backgeneration validation in 2020, and if the selected model continues to be used for forecasting it will possibly result in a large bias, so we counducted prediction by using the best model selected for each province excluding Hubei, and the incidence data for 2021–2024 were obtained for each province and city, as shown in Table 3. What’s more, the predicted incidence of Class B notifiable infectious diseases was visualised for each province and is shown in Fig. 6. The predicted incidence in most regions of China from 2021 to 2024 shows a decreasing trend year by year, while it shows the opposite in such regions as Guangdong, Hainan, Hunan, Guangxi, Guizhou and Tibet, whose predicted incidence rates of Class B notifiable infectious diseases in 2021–2024 range from 301.41/100,000 to 311.27/100,000, 375.67/100,000 to 436.99/100,000, 306.55/100,000 to 326.71/100,000, 303.16/100,000 to 307.43/100,000, 264.32/100,000 to 280.62/100,000, and 389.49/100,000 to 476.63/100,000 respectively. The overall incidence rate of Class B notifiable infectious diseases in China still shows a distribution pattern of high in the west and low in the east, but the difference between the east and the west is decreasing year by year.

**Table 3:** Fitting and prediction results of ARIMA model for the incidence of Class B notifiable infectious diseases by district

Region	Best model	MAPE(%)	Actual value	RE(%)	Forecast value				
			2020	2020	2020	2021	2022	2023	2024
Anhui	(0, 1, 1)	2.34	225.08	1.18	227.73	225.74	223.55	221.35	219.74
Beijing	(1, 1, 1)	3.11	80.70	9.43	88.31	81.69	75.44	70.12	65.58
Fujian	(1, 1, 1)	2.18	223.99	7.87	241.61	226.61	211.42	198.46	185.73
Gansu	(2, 1, 1)	1.47	150.52	1.22	152.36	128.79	110.47	96.53	78.58
Guangdong	(0, 1, 1)	2.98	272.49	1.72	277.19	301.41	305.21	308.19	311.27
Guangxi	(0, 2, 1)	4.76	263.62	2.57	270.40	303.16	305.60	306.45	307.43
Guizhou	(1, 1, 1)	3.90	248.35	4.01	258.32	264.32	269.17	275.41	280.62
Hainan	(0, 1, 1)	3.54	339.12	4.29	353.68	375.67	397.33	415.97	436.99
Hebei	(1, 2, 1)	2.18	134.26	8.49	145.66	138.68	134.28	129.73	125.71
Henan	(1, 1, 1)	2.11	152.77	2.26	156.22	149.60	134.98	120.36	105.74
Heilongjiang	(0, 1, 1)	5.34	105.84	7.35	113.62	110.54	106.46	101.37	96.29
Hubei	(1, 2, 1)	2.18	290.81	19.55	233.95	–	–	–	–
Hunan	(1, 1, 1)	2.76	269.01	3.65	278.83	306.55	313.27	319.99	326.71
Jilin	(1, 1, 0)	4.72	87.30	8.68	94.88	93.99	90.61	88.79	87.55
Jiangsu	(0, 1, 1)	3.11	98.23	5.48	103.61	96.12	91.53	86.85	81.07
Jiangxi	(0, 1, 1)	4.15	199.30	7.12	213.49	204.15	195.76	187.49	181.49
Liaoning	(0, 2, 1)	3.13	155.22	6.94	165.99	152.99	139.21	131.87	123.72
Neimenggu	(1, 1, 1)	6.11	225.64	7.78	243.19	232.16	221.12	211.09	195.05
Ningxia	(0, 1, 1)	2.18	168.75	6.15	179.13	161.17	148.23	131.27	117.32
Qinghai	(2, 1, 1)	4.81	376.80	6.84	402.58	398.67	388.58	382.72	377.18
Shandong	(1, 1, 1)	2.31	131.73	8.77	143.28	132.21	125.17	119.81	112.05
Shanxi	(0, 1, 1)	1.74	202.95	8.33	219.86	209.24	201.35	192.98	184.65
Shaanxi	(0, 1, 1)	2.73	150.48	7.87	162.33	154.35	148.41	142.45	138.49
Shanghai	(1, 1, 1)	2.28	128.63	7.04	137.69	131.03	124.27	118.52	112.77
Sichuan	(1, 2, 0)	1.37	196.03	3.91	203.69	195.03	188.27	180.52	172.77
Tianjin	(1, 1, 1)	2.43	109.28	9.18	119.31	105.11	92.04	83.48	72.01
Xizang	(0, 1, 2)	5.69	337.10	8.79	366.74	389.49	419.56	441.91	476.63
Xinjiang	(1, 1, 2)	4.82	324.99	8.75	353.42	341.57	329.14	313.63	294.12
Yunnan	(0, 2, 1)	4.51	189.61	9.04	206.75	201.90	195.03	190.16	184.29
Zhejiang	(1, 1, 1)	1.32	146.47	6.26	155.64	147.54	132.59	123.39	111.53
Chongqing	(1, 2, 0)	3.18	202.07	5.47	213.13	210.60	209.49	207.61	204.85

## Discussions

This study uses the incidence rates of Class B notifiable infectious diseases published on the official website of the National Health Commission of the People’s Republic of China from 2007 to 2020 as a data source to analyse the characteristics of the spatial and temporal distribution of the incidence rates of Class B notifiable infectious diseases in China, and to forecast their development trends. The results of the study show that, in terms of spatial distribution, the overall incidence of Class B notifiable infectious diseases in China during the period 2007–2020 showed an increasing distribution of “East-Central-West”, indicating that the incidence of Class B notifiable infectious diseases has a certain correlation with the level of economic development of the region. The incidence rate of Class B infectious diseases is correlated with the economic development level of the region. Previous studies have confirmed that higher levels of economic development can be beneficial to the reduction of likelihood of infectious disease outbreaks, as well as the disease hazards associated with infectious disease outbreaks [32]. For example, Wu et al. [33] explored the impact of climate change on human infectious diseases and found that developing countries face greater health risks from infectious disease outbreaks than developed countries because their public health systems lack the resources and capacity to respond effectively to the challenges. The present study confirms that the incidence of Class B notifiable infectious diseases is lower in the economically developed eastern region of China than the central and western regions, which is easily explained by the fact that the eastern region has more medical and educational resources and better infrastructure than the central and western regions, and will suffer less from the burden of disease caused by infectious diseases, especially vaccine-accessible infectious diseases such as measles, whooping cough and tuberculosis [34]. However, the incidence of Class B notifiable infectious diseases decreased the most in the west, and the regional differences in incidence rates was on a decreasing trend. The analysis suggests that although the western region is relatively lagging behind in economic development, it has benefited from the Western Development Policy in recent years, making it possible to achieve significant results in the prevention and treatment of infectious diseases in the western region. The identification of regional differences in the incidence of Class B notifiable infectious diseases is conducive to the adoption of differentiated infectious disease prevention and control intervention strategies for different regions. Since effective prevention and control of infectious diseases in the central and western regions is crucial to reducing the overall incidence of infectious diseases nationwide, it is much necessary to continue to increase the investment and talent introduction policies for the central and western regions. Through the “policy dividend” [35], the economic development of the central and western regions and the level of health prevention and control can be improved. Meanwhile, the eastern region, as the driving force in the improvement of infectious disease prevention and control, should interact well with the central and western regions and play a leading role in order to reduce the health hazards of infectious diseases to the Chinese population.

A lot of studies have shown significant spatial clustering in the incidence of Class B notifiable infectious diseases such as typhoid and paratyphoid fever, dengue fever and novel coronavirus [36–39]. According to the current stuy, the Moran’s *I* index for the incidence of Class B notifiable infectious diseases in China from 2007 to 2020 was significantly positive, indicating that the incidence of Class B notifiable infectious diseases showed a strong clustering in spatial distribution, which is consistent with the results of previous studies. Besides, the Moran’s *I* index values show an increasing trend year by year, indicating a gradual increase in the degree of clustering in the spatial distribution of the incidence of Class B notifiable infectious diseases. In other words, there is a spatial correlation between the incidence of Class B notifiable infectious diseases in each province of China and that in neighbouring provinces, and the degree of correlation is gradually increasing. This result confirms the fact that no provincial unit can control infectious diseases without cooperating with other provinces in the fight against them, and no region can protect itself from an infectious disease crisis alone. Therefore, in order to reduce the incidence of Class B notifiable infectious diseases in a particular province, the level of economic development and the level of sanitary and epidemiological protection in the province as well as in the surrounding provinces need to be taken into account as important factors affecting the effectiveness of infectious disease prevention and control [40–42]. What’s more, in order to reduce the overall incidence of Class B notifiable infectious diseases in China, it is recommended that a regional community for improvement in infectious disease prevention and control be formed by breaking down geographical and administrative barriers and delineating a multi-level regional framework, and that inter-provincial mechanisms for joint prevention and control of infectious diseases be established so as to give full play to the positive spatial spillover effects of the positive prevention and control of infectious diseases across the region.

According to the incidence rates of Class B notifiable infectious diseases in China from 2007 to 2020, the national overall rates as well as the rates in each province are decreasing except a certain rise in four provinces (Guangdong, Hainan, Hunan, and Tibet). This indicates that we have achieved notable results in the prevention and control of infectious diseases in China, which is consistent with the results of previous studies [43]. According to the research analysis, the fact that the four provinces have not seen a decline in the incidence of Class B notifiable infectious diseases may be mainly related to their special geographical location and climatic conditions [44–49]. In spite of a certain economic boost in Tibet, a border region in the southwest, the increasing trade flows results in the bigger size of the mobile population, which leads to the problem of new and traditional infectious diseases and their increasingly prominent cross-border transmission. Guangdong, Hunan and Hainan all have tropical or subtropical monsoon climates, which is temperate and conducive to the prevalence of climate-sensitive mosquito-borne viral diseases such as dengue, malaria and encephalitis B.

After verification and adoption of the selected best model using the data of 2020, the short-term forecast for 5 years from 2020 to 2024 was carried out using the best model. The results show that, firstly, compared with the predicted 2020incidence assuming no COVID-19 epidemic broke out, the actual 2020 incidence of Class B infectious diseases are smaller for both China as a whole and all the provinces (except Hubei Province), suggesting that the prevention and control measures taken in response to the COVID-19 epidemic are conducive to controlling the occurrence and development of other Class B notifiable infectious diseases, which is consistent with the results of existing studies [50–52]. The study suggests that it is the government’s mandatory prevention and control strategies as well as the public’s increasing awareness of personal health that works. On the one hand, studies have shown that non-pharmaceutical interventions implemented by the government during the COVID-19 epidemic (e.g. school closures, movement restrictions and social distancing) contributed to a decline in the incidence of infectious diseases such as pertussis, scarlet fever and hand, foot and mouth disease (HFMD) [53]. On the other hand, both the initial period of rigorous epidemic prevention and control and the subsequent period of normal practice of them saw an increase in public awareness of personal health. Such intervention measures as wearing masks, social distancing, hand washing and ventilation also effectively prevent the spread of other infectious diseases transmitted through respiratory tract, intestinal tract or intimate contact, for example, whooping cough, scarlet fever, tuberculosis, and brucellosis, etc. Secondly, the forecast of the incidence of Class B notifiable infectious diseases in China from 2021 to 2024 shows that the incidence of infectious diseases is still on a downward trend nationwide and in most provinces, but the predicted incidence of Class B notifiable infectious diseases in such provinces as Guangdong, Guizhou, Hunan, Hainan, Tibet and Guangxi is on an upward trend, which suggests that the government should focus more attention upon provinces where the incidence of infectious diseases is on an upward trend, and tailor specific measures to the actual situation of each province to avoid the re-emergence of certain infectious diseases in a province or even nationwide and the outbreak of new infectious diseases due to relaxed vigilance. The ARIMA model is highly accurate (within 10%) in predicting the incidence of Class B notifiable infectious diseases, and can effectively compensate for the current lack of capacity to develop, evaluate, manufacture, distribute and manage effective medical countermeasures (e.g. vaccines, diagnostics, etc.), effectively address the unmet disease burden associated with outbreaks or prevalence of traditional and emerging infectious diseases, and effectively guide policy decisions such as rational allocation of health resources and pre-deployment of emergency supplies [54].

There are, of course, certain limitations in this study. The first limitation is about the prediction accuracy of the ARIMA model. On the one hand, the prediction accuracy of the ARIMA model is easily affected by the sample size. The bigger the sample size, the higher the prediction accuracy of the model, with the number of variables unchanged. In this study, based on the principle of indicator data availability, 13 years of data (2007–2019) were finally selected for fitting the ARIMA model, using 2020 data for model validation and forecasting the incidence of Class B notifiable infectious diseases in China as well as in each province from 2021 to 2024. Although the relative errors of the selected models were less than 10% for China as a whole and for each province (except Hubei Province), the prediction accuracy could be further improved in the future by increasing the sample size. On the other hand, it is worth mentioning that while this study recognises that notifiable infectious diseases in Class B are more infectious and may lead to outbreaks or epidemics with relatively high sensitivity and specificity compared with notifiable infectious diseases in category C. The use of incidence data for Class B notifiable infectious diseases can effectively reduce the reduction in predictive accuracy of ARIMA models due to missing reports. However, it is worth mentioning that the prediction results may deviate from the actual values over time due to the changing external environment of the host, e.g. policy instability and vaccine availability [55]. Therefore, it is much necessary to update the forecast of the incidence of Class B notifiable infectious diseases in accordance with the availability of data. Secondly, this study uses the annual incidence of Class B notifiable infectious diseases, a multi-disease joint data, for the prediction study. While this is good for getting a full picture of infectious diseases, it also has shortcomings because even if problems can be identified, it still takes time to determine the specific situation. Future applications will need to be tailored to the specific characteristics of each disease. Finally, the study on the incidence of Class B notifiable infectious diseases is mainly an exploratory spatial data analysis. Future studies can be made on the basis of the existing studies by combining other variables such as economic factors, transport factors and population mobility factors to conduct empirical spatial data analysis through the establishment of spatio-temporal regression models and to explore the causes and driving mechanisms of the formation and changes in the spatio-temporal patterns of the incidence of Class B notifiable infectious diseases.

## Conclusion

This study introduced GIS spatial statistics into the study of infectious diseases, and revealed the spatial association patterns and spatio-temporal evolution characteristics of Class B notifiable infectious diseases in China at a macro level. During the period 2007–2020, infectious disease prevention and control in China has been effective in a certain degree, but there is a clear “east-central-west” decreasing geographical difference in prevention and control effectiveness. The spatial distribution of the incidence rate of Class B notifiable infectious diseases in China is clustered and the degree of clustering is increasing year by year. This study also demonstrates that the ARIMA model can be used to predict the incidence of Class B notifiable infectious diseases, a multi-disease joint data. The results of this study will not only help to facilitate the application of geographic methods to health care, but assist in resource allocation and preparedness planning by accurately predicting the potential geographic extent of disease incidence and transmission of infectious diseases, providing effective information for evidence-based scientific prevention and control of infectious diseases.

## Supplementary Information

**Additional file 1.** The classification of reported infectious diseases in China.

## Data Availability

The original data can be found on the National Health Commission of the People’s Republic of China (http://www.nhc.gov.cn) and the standard map service website of the National Bureau of Surveying, Mapping and Geo-graphic Information (http://bzdt.ch.mnr.gov.cn).

## Abbreviations

COVID-19: Coronavirus disease 2019
SARS: Severe acute respiratory syndrome
GIS: Geographic information system
ARIMA: Auto regressive integrated moving average
BIC: Bayesian information criterion error
RE: Relative error
MAPE: Mean absolute percentage error
ACF: Autocorrelation function.
PACF: Partial autocorrelation function.
