# Supplementary material for: Spatio-temporal evolution and trend prediction of the incidence of Class B notifiable infectious diseases in China: a sample of statistical data from 2007 to 2020
Source: BMC Public Health. 2022 Jun 17;22:1208. doi: 10.1186/s12889-022-13566-2 (PMC9204078; doi:10.1186/s12889-022-13566-2)
Supplement: Supplementary file 1 — Additional file 1. The classification of reported infectious diseases in China. [file 12889_2022_13566_MOESM1_ESM.docx]

Annex 1

**Additional file 1** The classification of reported infectious diseases in China

| **Classification** | **Included infectious diseases** |
| --- | --- |
| **Class A** | Plague, Cholera; |
| **Class B** | Severe Acute Respiratory Syndrome (SARS), Acquired immunodeficiency syndrome (AIDS), Viral hepatitis, Poliomyelitis, Human infections of highly pathogenic avian influenza, human infections of H7N9 avian influenza, Measles, Epidemic hemorrhagic fever (EHF), Rabies, Epidemic encephalitis B, Dengue, Anthrax, Bacterial and amoebic dysentery, Tuberculosis, Typhoid & paratyphoid , Epidemic (meningococcal) meningitis, Pertussis, Diphtheria, Neonatal tetanus, Scarlet fever, Brucellosis, Gonorrhea, Syphilis, Leptospirosis, Schistosomiasis, Malaria, Human H7N9 Avian Influenza, Coronavirus Disease 2019 (COVID-19); |
| **Class C** | Influenza, Mumps, Rubella, Acute hemorrhagic conjunctivitis (AHC), Leprosy, Typhus, Leishmaniosis, Echinococcosis, Filariasis, Other infectious diarrheal diseases, Hand, foot and mouth disease (HFMD). |
